# Blockchain-based cloud manufacturing platforms: A novel idea for service composition in XaaS paradigm

**DOI:** 10.7717/peerj-cs.743

**Published:** 2021-12-08

**Authors:** Seyyed-Alireza Radmanesh, Alireza Haji, Omid Fatahi Valilai

**Affiliations:** 1 Advanced Manufacturing Laboratory, Industrial Engineering Department, Sharif University of Technology, Tehran, Tehran, Iran; 2 Department of Mathematics & Logistics, Jacobs University Bremen, Bremen, Bremen, Germany

**Keywords:** Cloud manufacturing, Blockchain technology, Service composition, Proof of optimality

## Abstract

Cloud manufacturing is a new globalized manufacturing framework which has improved the performance of manufacturing systems. The service-oriented architecture as the main idea behind this framework means that all resources and capabilities are considered as services. The agents interact by way of service exchanging, which has been a part of service composition research topics. Service allocations to demanders in a cloud manufacturing system have a dynamic behavior. However, the current research studies on cloud-based service composition are mainly based on centralized global optimization models. Therefore, a distributed deployment and real-time synchronization platform, which enables the globalized collaboration in service composition, is required. This paper proposes a method of using blockchain to solve these issues. Each service composition is considered as a transaction in the blockchain concept. A block includes a set of service compositions and its validity is confirmed by a predefined consensus mechanism. In the suggested platform, the mining role in blockchain is interpreted as an endeavor for proposing the proper service composition in the manufacturing paradigm. The proposed platform has interesting capabilities as it can increase the response time using the blockchain technology and improve the overall optimality of supply-demand matching in cloud manufacturing. The efficiency of the proposed model was evaluated by investigating a service allocation problem in a cloud manufacturing system in four large scale problems. Each problem is examined in four centralized modes, two, three and four solvers in blockchain-based model. The simulation results indicate the high quality of the proposed solution. The proposed solution will lead to at least 15.14% and a maximum of 34.8 percent reduction in costs and 20 to 68.4 percent at the solving time of the problem. It is also observed that with increasing the number of solvers (especially in problems with larger dimensions) the solution speed increases sharply (more than 68% improvement in some problems), which indicates the positive effect of distribution on reducing the problem-solving time.

## Introduction and problem definition

### Cloud manufacturing definition

Nowadays, modern manufacturing systems are struggling to fulfill the requirements of global supply chain models (Meixell & Gargeya, 2005; Supriya & Djearamane, 2013). Of these dominant systems, cloud supply network is a new service-oriented model based on concepts and technologies such as cloud manufacturing, Internet of Things, Industry 4.0, and Big data, that enables the sharing and collaboration of supply resources over the globe (Valilai & Houshmand, 2014a; Valilai & Houshmand, 2014b).

**Figure 1:**
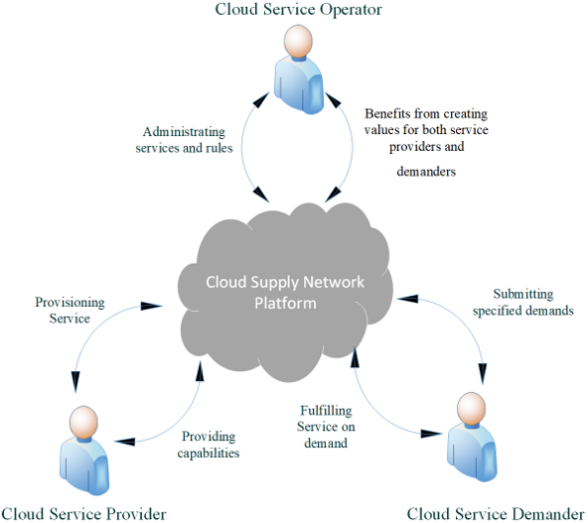
The overall structure of cloud manufacturing.

Considering the merits of cloud manufacturing paradigm as an everything-as-a-Service (XaaS) approach, it can be extended towards the cloud supply network. Using this approach, a general abstract model for a cloud manufacturing system can be visualized as shown in Fig. 1. The cloud customers or service demanders submit their demands to the cloud platform and the cloud service providers fulfill the service demands provisioned by the cloud operators. Cloud operators provide different services generally grouped as administrative services, and receive benefit from creating values for both service providers and demanders. As the XaaS approach considers all manufacturing operations as services distributed over the globe, logistics operations must be enabled among manufacturing operations. So, the XaaS approach can be considered to encompass the logistics operations as services resulting to a global cloud supply network (Aghamohammadzadeh, Malek & Valilai, 2019). The research studies on architectures and platforms for cloud manufacturing can contribute to enabling the cloud supply network architectures that encompass operational requirements and logistics considerations (Aghamohammadzadeh & Valilai, 2020; Rezapour Niari, Eshgi & Fatahi Valilai, 2021).

Among the most challenging research issues in cloud manufacturing are service composition, task scheduling and transportation planning (Zhou & Yao, 2017). Service composition is one of the most important issues in cloud manufacturing and deals with discovering, allocating, and scheduling logistics and operational services in the resource pool to supply requirements (Delaram et al., 2021a; Delaram et al., 2021b).

#### Cloud manufacturing challenges

The main feature of the cloud manufacturing system is its service-oriented architecture. Every resource or capability is a service provider in the cloud network. One of the key roles of cloud platform administration is the proper allocation of services and demands while fulfilling operational constraints. Considering the changes in demand and service and new service definitions in the cloud, allocating services to demanders in a cloud manufacturing has a dynamic behavior and will depend on previous allocations, current network capabilities, and newly generated demands. Therefore, a dynamic approach is needed in service composition. Current research studies on cloud-based service composition are mainly based on centralized global optimization models. However, the dynamic characteristic of the problems and their size in a globalized cloud manufacturing system will challenge the efficiency of centralized solutions. Therefore, distributed architectures with real-time enabled synchronization platforms are needed to enable the globalized collaboration among manufacturers.

This paper proposes a strategy for transferring from a central current state-of-the-art mechanism to distributed management and planning model in cloud manufacturing. This strategy will solve the abovementioned challenges by spreading computational capabilities over the network. A decentralized cloud manufacturing system has many advantages over the central model. For example, decentralization will greatly reduce the costs and a distributed system will have faster transactions and agile service compositions. A decentralized platform for solving dynamic service composition challenges increases efficiency for service demanders. This is accomplished by allowing more parties to collaborate and compete with each other for service composition and using available resources. Data on the network service and demand in cloud pools will be transparent, safe, irreversible, and possible data failures will be minimized.

### Blockchain technology

Blockchain is a distributed and decentralized database of transactions promulgated among nodes in a network. Each transaction or record in this database should be verified by a consensus mechanism that is executed by the major participant’s nodes (Crosby et al., 2016). Blockchain is not managed by a central entity but rather by an autonomous peer-to-peer network. Therefore, the challenges of central management are eliminated in this protocol, and the decentralized mechanism of blockchain improves security, scalability, transparency, and agility. Furthermore, records in blockchain are irrefutable and fraud or counterfeiting events are nearly impossible in this mechanism. Due to these advantages, this technology has a large potential application beyond cryptocurrencies and in fields like healthcare, education, supply chain, and governance.

A blockchain is formed by a chain of blocks that are linked together in a proper and chronological order. Each block consists of a group of records or transactions that are completed simultaneously. The enthusiastic nodes in this network—named miners—create these blocks through a predefined consensus mechanism. The most famous consensus mechanism is proof of work which is used in Bitcoin blockchain. According to this consensus method, miners should solve a special mathematical puzzle to create a valid block. For example, in the Bitcoin network, miners should guess a nonce. When this nonce is hashed in a defined manner, it produces a hash with a certain number of leading zeroes (Nakamoto, 2008). After a block is validated by the blockchain consensus mechanism, it should be promulgated in the network to be replicated in all other miners’ ledger. This way, valid transactions can be publicly stored in the database. The longest blockchain is the valid one and is accepted by the whole network. So, if an attacker wants to alter a prior transaction or introduce a fraudulent transaction in the blockchain, they must solve some mathematical puzzles to generate a block containing this transaction and subsequent blocks to create the longest blockchain. In reality, it is only possible if a single attacker’s computational power is greater than half of the authentic miners in the network. This makes double-spending in the blockchain impossible (Crosby et al., 2016).

In this study, the service allocation problem in the cloud manufacturing and supply framework has been solved with higher efficiency by incorporating the blockchain technology. From the quantitative aspect, the proposed architecture has decreased the required solving time while simultaneously improving the efficiency. From the qualitative aspect, the proposed architecture has succeeded in solving several problems, including a central entity making decisions for the whole system regardless of the preferences of each system unit, scalability limitations, and single point of failure, all of which are explained in detail in the following sections.

This paper aims to enable a model using blockchain technology for cloud manufacturing service composition. The rest of the paper is structured as follows. In ‘Literature review and related research studies’, literature review and an investigation of related research studies are presented. In ‘The proposed platform for cloud manufacturing using blockchain mechanism’, inspired by the blockchain concept, the proposed decentralized framework for cloud manufacturing system is illustrated and its related technical concept mappings to the blockchain paradigm are discussed. ‘Numerical study for the capabilities of the proposed platform’ investigates numerical examples to demonstrate the proposed framework capabilities. Finally, in ‘Discussion and conclusion’, the conclusions and future research are discussed.

## Literature review and related research studies

In recent years, service-oriented or everything-as-a-service (XaaS) models have received a great deal of attention from both academia and the industry (Liu & Xu, 2017). XaaS models have many advantages in manufacturing systems. For instance, the distributed manufacturing resources and capabilities could be used by a centralized management based on demand (Zhang et al., 2010). XaaS architecture can acquire services for scalable and economical resource sharing and coordinated collaboration. In the XaaS architecture, on-premise-related costs like software, hardware, and maintenance are eliminated and the productivity will be increased by the better utilization of Information Technology advantages (Adamson et al., 2017). Companies in the XaaS paradigm that use the economy-of-scale would have significant advantages over competitors (Manenti, 2011).

Sharing resources in the cloud manufacturing network enhances the business agility and fulfills the globalization paradigm (Houshmand & Valilai, 2013; Valilai & Houshmand, 2014c). Cloud manufacturing aggregates distributed manufacturing resources and constructs a network of the manufacturing resources (Ren et al., 2015) and, therefore, enables a wide network of manufacturing capabilities to simultaneously deal with tasks with different requirements. In recent years, manufacturing systems require agility and ubiquity in terms of operation flexibility due to the importance of customization, personalization, and individualization (Delaram & Valilai, 2016; Delaram & Valilai, 2018; Strandhagen et al., 2017). Therefore, cloud manufacturing has the potential of changing the production-oriented environment into a service-oriented environment (Delaram & Valilai, 2017). As stated earlier, service composition is one of the challenging issues in cloud manufacturing system. It refers to the discovery, allocation, and scheduling of logistics and operation services in the resource pool to supply requirements in the order demand pool. In Table 1, recent research studies on service composition are presented. The major gap in the literature is the lack of mechanisms for fulfilling the dynamic service and demand behaviors. Also, all the research studies are limited as they consider a centralized mechanism for service composition administration. As the cloud network grows and the dynamic parameters are considered, the centralized mechanism loses its efficiency for an agile and efficient service management.

**Table 1:** Recent research studies on service composition.

Researchers	Description
Tong & Zhu (2020)	Proposed a two-layer social network model for service composition from the viewpoint of synergy.
Aghamohammadzadeh, Malek & Valilai (2019)	Logistics services have been considered as shared services and the authors proposed an optimal solution for manufacturing and logistics service composition.
Yuan et al. (2020)	A multi-task corresponding multi-service selection problem was studied and an optimization problem that considers six Quality of Services has been solved.
Yang et al. (2019b)	In this paper, energy-aware service composition has been addressed and low-energy consumption has been considered as a Quality of Service function in a green and sustainable manufacturing system.
Yang et al. (2019a)	A new dynamic ant-colony genetic hybrid algorithm has been proposed to solve the large-scale cloud service composition and optimization.
Bouzary & Chen (2019)	The service composition-optimal selection problem has been solved by the grey wolf optimizer algorithm and evolutionary operators of the genetic algorithm.
Fazeli, Farjami & Nickray (2019)	In this paper, the Ensemble optimization approach has been proposed to solve the service composition problem in cloud manufacturing and its result shows a better solution compared with the genetic algorithm.
Zhou et al. (2018)	An improved artificial bee colony algorithm by introducing a synergetic mechanism for food source perturbation has been proposed to solve many-objective service composition problems.
Zhang et al. (2018)	An extended flower pollination algorithm employed to solve the service composition problem and efficiency of the suggested method was compared with the genetic algorithm.
Li et al. (2017)	The service clustering network-based service composition approach has been addressed and the efficiency of the proposed approach has been verified by simulation experiments.
Ahmadi et al. (2021)	The blockchain technology has been considered to propose a conceptual framework to enable the collaboration of multiple suppliers and vendors together.

Blockchain was introduced for the first time by an unidentified individual or a group of individuals called Satoshi Nakamoto in 2008, as part of a proposal for Bitcoin entitled “*Bitcoin: A peer-to-peer electronic cash system*” (Nakamoto, 2008). Seebacher & Schüritz (2017) proposed a structured and systematic procedure for assessing Blockchain technology characteristics. These characteristics include trust, being shared and public, low friction, peer verification, cryptography, immutability, decentralization, pseudonymity, redundancy, versatility, and automation.

Some reserches presented three main types of blockchain depending on diverse applications (Francisco & Swanson, 2018). In blockchain type 1, the concept of cryptocurrencies was developed. Cryptocurrencies like Bitcoin are of this type. Beyond cryptocurrencies, the researchers denoted smart contracts in which blockchain 2 technology is considered. Some potential applications of this type include financial transactions, public records, cloud storage, crowdfunding, and private blockchains. The third and last blockchain type was proposed to be useful in governance, health systems, and sciences. Blockchain-based applications in various industries like supply chain and manufacturing are derived from blockchain type 2.

The paper has also investigated the application of blockchain technology in supply chain and manufacturing. The research studies indicate that many challenges exist in a traditional supply chain management system and manufacturing system which can be solved in blockchain-based mechanisms. One of the important issues in supply chain that can be effectively resolved with blockchain is the capability of safe and transparent tracking of goods and materials. Kim & Laskowski (2018) proposed an ontology-driven blockchain architecture for supply chain to determine the provenance of goods by considering the big data in a supply chain. Enhanced trust through transparency and its potential benefits in the manufacturing system have been discussed in the literature (Apte & Petrovsky, 2016). Furthermore, a visionary for the future blockchain enhanced manufacturing system, its requirements, and challenges for adoption in the future manufacturing systems were discussed.

A review on how blockchain could reshape supply chains addressed in Wang, Han & Beynon-Davies (2019). Three main benefits of blockchain to supply chain are presented in this research. The main benefit is improving supply chain visibility and transparency. Other important benefits of Blockchain are irreversibility and immutability. In some types of manufacturing systems, central mechanisms are developed to monitor and create transparency among manufacturing entities. A conceptual design for supply chain traceability based on the Unified Theory of Acceptance developed in Francisco & Swanson (2018). An implementation of an Ethereum-based application for tracking parcels in a supply chain designed by Helo & Hao (2019).

A blockchain-based service composition architecture to enhance information transparency and decentralization in the cloud manufacturing system (Yu et al., 2020). The main challenge discussed in this paper is the information possessing by a central entity. Another benefit mentioned in Wang, Han & Beynon-Davies (2019) secure information sharing and building trust. The last benefit is allowing for operational improvements; for example, blockchain speeds up end-to-end manufacturing execution. However, application of the blockchain as the facilitator of service composition in cloud-based manufacturing has been ignored by the blockchain technology researchers.

As mentioned above, blockchain technology has many applications in cloud systems and cloud manufacturing. In Table 2, recent research studies on blockchain technology application in the cloud manufacturing has been addressed.

**Table 2:** Recent research studies on Blockchain technology applications in the cloud manufacturing.

Reference	Main Idea	Summary
Hasan & Starly (2020)	They proposed a blockchain-based architecture to connect customers and service providers directly.	In this paper, a blockchain-based architecture to remove the middle-man from the cloud manufacturing-as-a-service platforms is introduced. The design results in improving transparency, data integrity, data provenance, and retaining data ownership to its creators. It is implemented on Ethereum blockchain, and its performance is evaluated and compared with other central systems.
Yu et al. (2020)	They proposed a blockchain-based cloud manufacturing architecture	They suggested a blockchain-based cloud manufacturing architecture that aims at improving transparency and decentralization. It assumes production resources as a service traded and distributed on the blockchain. The quality-of-service composition is measured, and by employing the metaheuristic swarm optimization (PSO) model, the distribution results are estimated.
Aghamohammadzadeh & Valilai (2020)	It introduce a specified architecture in cloud manufacturing to improve the service composition	The problem of decentralized service composition in cloud manufacturing is attempted to be addressed in this paper. The proposed model subdivides the problem based on geographical locations where a miner solves each sub problem.
Zhu et al. (2020a) and Zhu et al. (2020b)	It proposes a novel pricing model for cloud manufacturing based on the blockchain	Applying the game theory and fuzzy algorithm, a pricing model is introduced. In the proposed framework, blockchain is used to solve the pricing problem for cloud manufacturing.
Vatankhah Barenji (2021)	It introduces Blocktrust model to enhance trust in the cloud manufacturing system	How the supplier and customer trust each other in a cloud manufacturing system is an ongoing research topic. It is common to solve this issue by mechanisms developed by the central authority, which may lead to a high cost. This paper attempts to solve the problem without any central authority involved. The model is implemented on the private Hyperledger blockchain, and the results are presented.
Zhang et al. (2021)	It introduces a consensus mechanism named proof of service power	They suggest a consensus mechanism named proof of service power, a practical mechanism for cloud manufacturing based on blockchain. This consensus mechanism suits cloud manufacturing more than the standard ones in the literature, like proof of stake and proof of work. Moreover, less energy is consumed, and more transactions are stored. Service power represents each customer or supplier history.
Zhu et al. (2020a) and Zhu et al. (2020b)	They propose a consensus-oriented cloud manufacturing based on the blockchain technology	This paper tries to answer the common problems of cloud manufacturing like trust, transparency, and payment by a blockchain-based architecture. Proof of authority consensus mechanism is applied to simulate the architecture, and the final results are presented.
Li et al. (2019), Vatankhah Barenji et al. (2018) and Vatankhah Barenji et al. (2020)	They propose a blockchain-based platform as a trustable network to eradicate third-party problems,	A blockchain-based model is proposed for centralized cloud manufacturing to solve problems related to trust, scalability, and big data.
Li et al. (2019)	It proposes a trust mechanism based on the blockchain technology	Considering the blockchain structure that leads to decentralized data saving and invariable storage, blockchain data is secure and well-guarded. This paper attempts to employ the mentioned blockchain characteristics to solve the related issues of cloud manufacturing.

As shown in the Table 2, the number of researchers attempting to apply blockchain technology to cloud manufacturing in recent years has risen remarkably. Nonetheless, most of the literature is limited to placing trust among the various parts of a cloud manufacturing system or proposing an architecture to apply blockchain technology to cloud manufacturing. The number of research studies aiming to introduce a new consensus mechanism tailored to solve the optimization problems excited in cloud manufacturing is minor to the authors’ best knowledge. This paper plans to address the exciting gap in the literature by proposing a consensus mechanism to solve optimization problems such as service composition in cloud manufacturing as well as architecture. Indeed, the proposed design attempts to answer the previous papers’ concerns like trust, transparency, big data, etc.

According to the literature review, the following shortages in the related research studies have been identified:

- In most research studies, cloud manufacturing has two distributed sides, service providing and consuming, and one central side, service composition. The planning and solving tasks are not distributed and can benefit from decentralization.
- There are few research studies on the utilization of blockchain as a distributed model in manufacturing systems.
- A consensus mechanism for the service composition in cloud manufacturing system has not yet been proposed in the literature.

## The proposed platform for cloud manufacturing using blockchain mechanism

### Blockchain principles and characteristics

As mentioned in the related researches, five basic principles addressed in blockchain technology (Iansiti & Lakhani, 2017). First, blockchain is a distributed database with no central control of data flow. Second, transmissions are investigated in a peer-to-peer manner with no intermediary. Third, due to the replication process in the network, there will be full transparency with anonymity. Fourth, a consensus mechanism creates irreversibility of transactions or records which are entered in a verified block. Last, due to computational logic in the system, users can insert records among participants in an automatic procedure.

### The necessity and significance for the use of blockchain for cloud manufacturing system

In the real world, the service composition problems should be solved in a very short time to take advantage of the proposed solution especially considering dynamic behaviors of service providers and demanders. In a central architecture, to solve this large-scale problem, an enormous amount of processing time and cost is required, while in the decentralized or distributed architectures, a feasible or near-optimal solution can be obtained in a more agile and efficient way. In the proposed solution, solvers play the role of miners in the Bitcoin’s blockchain. The solvers should enter into a pre-defined competition to solve a sub-problem of service composition and propose their recommended solutions. Since a large number of solvers are collaborating to find a near-optimal solution, it is expected that the speed of obtaining a near-optimal solution will decrease compared to the central mode.

Moreover, even if it is assumed that processing time and cost are not critical issues, there is still a challenge for non-imposing preferences in a cloud-based supply chain. In the central mode, the most powerful entity in the supply chain may impose its intentions on the whole chain. But in the proposed blockchain-based architecture, miners (solvers) act as a third party and take global goals into account. So, miners should solve problems globally and the dominancy of preferences issue would be eliminated in the cloud supply chain paradigm.

In addition to the aforementioned motivations, the consensus mechanism in blockchain has unique advantages in cloud manufacturing. In reality, a consensus mechanism should guarantee the dynamism of cloud manufacturing. If the solution announced by a miner is accepted by the network, other miners would accept the suggested solution and update the new conditions of their problem and start working on the rest of the dynamic service composition alternatives.

**Figure 2:**
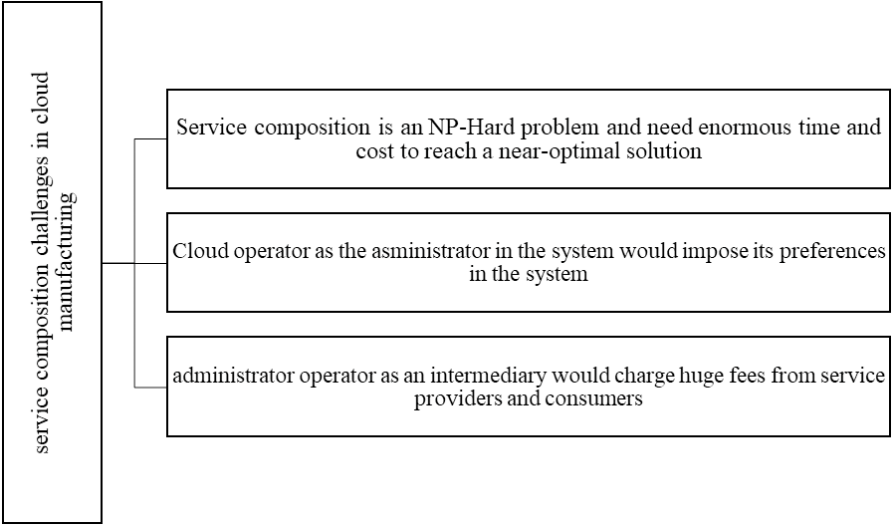
Service composition challenges in a central cloud manufacturing.

The main challenges of the central service composition have been summarized in Fig. 2.

### The proposed platform

In this paper, a new blockchain-based peer-to-peer distributed cloud manufacturing platform is proposed that allows eager participants to collaborate and suggest feasible and sufficiently optimal service compositions while considering global system conditions. This model empowers each business practitioner or expert to use their computational infrastructure, optimization algorithms, and intellectual capitals in the platform to suggest service composition solutions for service providers and demanders in the cloud manufacturing system.

To characterize the architecture of this system, first the cloud manufacturing planning should be redefined and ascertained to the underlying concepts in a blockchain paradigm.

### Transaction or record definition

Since Blockchain is a distributed ledger/database, this paper first specifies records or transactions of this database in a cloud manufacturing system. Each transaction should demonstrate global blockchain changes and transformations. Based on this context, service compositions are the main focus that should be determined and stored in a cloud manufacturing system. In each period, a collection of customer demands should be fulfilled with a collection of service providers’ capabilities. Since previous allocations and new system capabilities and demands have significant effects on achieving good service composition solutions, the proposed solution should be capable of validating and appending sets of service compositions that provide a near-optimal QoS objective function in the cloud manufacturing system. The utility of each service composition is determined by cloud manufacturing objective, which can be demand fulfillment time/rate or cost minimization and profit maximization. These objectives would be designated endogenously by network stockholders’ or exogenously by the cloud platform owner.

## Block definition

Each blockchain is a sequence of blocks that are linked together in a logical and chronological order. A block contains a collection of valid transactions or records, the hash output of the previous block (that is, the root of Merkle tree), and block header. In the proposed platform, a block consists of a set of service compositions that are created by allocating definite services to demands based on conditions like feasibility and local optimality. The objective function of a block depends on preceding blocks and its suggested service compositions.

## Mining and miners

Another important element in the blockchain paradigm is the miner. The miner, based on a specific protocol, guarantees the validity of the entire system transactions and records. A miner is an eager entity who first endorses acceptable and feasible records to be stored in blockchain and then, by fulfilling consensus mechanism requirements, creates a new block. In the proposed platform, each miner (solver) should propose a feasible set of service compositions and try to create better allocations of service compositions according to the objective function of the platform. When a miner (solver) creates a proper block, it will broadcast the block in the network and if it does not violate the former service compositions, the proposed block will be attached to the chain. When a new block is attached to the blockchain, other miners (solvers) should modify or restart their calculations in accordance with the attached block service composition solution.

**Table 3:** The proposed mapping of solver role to traditional Blockchain miners.

Comparison criteria	Miner- Bitcoin blockchain	Solver- proposed blockchain
Key role in the blockchain	-Verifying transactions -Creating a block through proof of work consensus mechanism	-Proposing service compositions for a sub-problem -Creating a block through proof of optimality consensus mechanism
Compensation model	-A transaction fee in each confirmed transaction -Fixed reward of creating each block	-A dynamic reward based on goodness of proposed service compositions through a pre-defined function
Required resources	-GPU for guessing a nonce to solve a mathematical puzzle in proof of work mechanism	-Proper optimization algorithms to solve the service composition problem -CPU to solve service compositions
Candidates for participation	-Everybody with a GPU!	-Everyone could enter the system but expects O.R. experts to be more eager for participating

In Table 3, a detailed comparison between miners (especially, miners in the Bitcoin blockchain) and solvers (the miner role in the proposed blockchain) has been illustrated.

## Consensus mechanism

A novelty in Bitcoin was the definition of a mechanism to prevent fraud and malicious attacks. In centralized systems, this protection is enabled by a trusted third party. But in Blockchain, many willing participants keep the system safe themselves without the presence of a single central entity. This mechanism is known as the consensus mechanism and has various types and applications in cryptocurrencies, such as proof of work (Antonopoulos, 2014), proof of stake, delegated proof of stake (Garay & Kiayias, 2020), on Byzantine consensus, and Proof of Activity (Sankar, Sindhu & Sethumadhavan, 2017) in blockchain paradigm. Reaching a consensus in a decentralized system was an important issue in distributed computing before the development of blockchain (Lakshman & Agrawala, 1986). One of the main advantages of blockchain is that it enables all nodes to reach consensus on reliability and validity of transactions and records. In the proposed blockchain-based cloud supply network platform, a specific proof of work mechanism is designed where each miner strives to, optimization methods, solve a mathematical problem containing the set of its service compositions. If, based on the consensus mechanism, the miner’s solution deserves reward (according to the incentive procedure) and is acceptable for becoming attached to the blockchain, it will be identified as a new block. The proposed consensus mechanism in the blockchain-based cloud manufacturing system encourages the miners’ proper release of service compositions. This paper considers this mechanism as the proof of optimality.

## Incentive procedure

**Figure 3:**
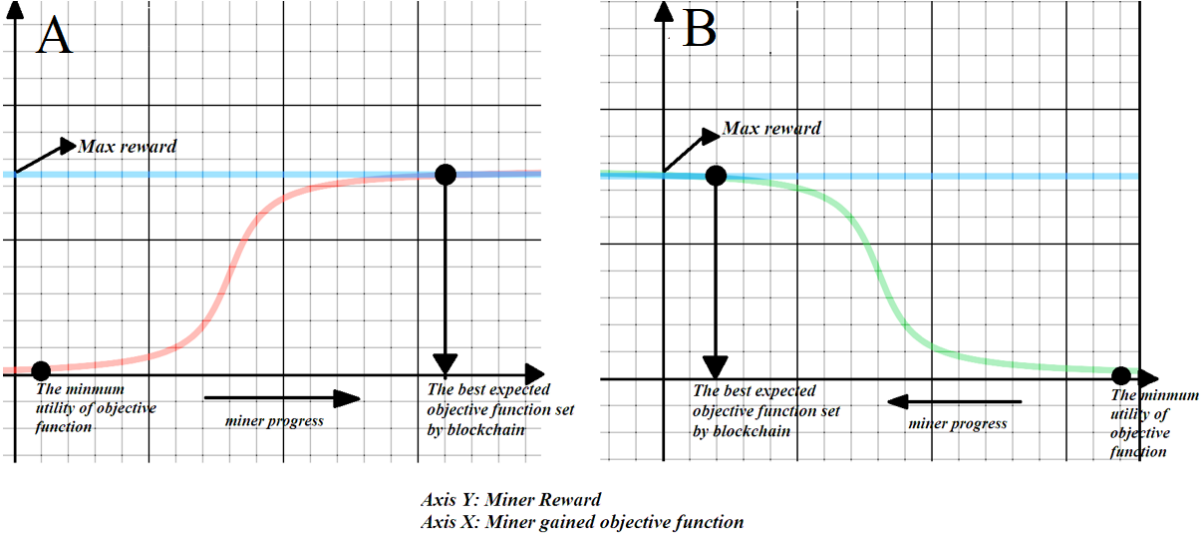
The schematic model of reward for both maximization and minimization objective functions. (A) Maximizing objective function, (B) minimizing objective function.

In blockchain, financial benefits are distributed among miners to encourage them to continue transaction verification and extending the blockchain. Each miner who satisfies network requirements in a correct and predefined procedure, deserves to get rewarded by the network. Once a block is generated, a number of coins will be created as an incentive and will be given to the winning miner. In the proposed platform, a reward is defined as an objective function of the optimality of the service composition set (Fig. 3). The miners compete with each other to propose valid, optimal sets of service compositions. Based on the tradeoff between increasing the optimality of their proposed set of service compositions or stopping and announcing their sets of service composition, the winning miner will benefit from the reward.

At the first step, a feasible set of service compositions are suggested by the network or cloud platform owner/administrator as the minimum utility, and an upper bound as the best guess of the magnitude of objective function. Proposed sets that have a lower utility than the proposed minimum solution will gain zero rewards and their block will not be accepted to be linked to the previous blocks in the chain. As shown in Fig. 3, if the reward schema is only a small improvement from the minimum solution, it will create a negligible reward and none of the miners will be motivated to announce this solution. But a sufficiently better solution than the minimum would have a much greater reward. Therefore, miners compete both for faster finishing and announcing their block and having a better utility in their sets of service compositions. High greedy approaches for achieving the optimal service composition set will result in zero revenue as the other competing miners will tradeoff, and announce their service composition set as block that, if it does not violate the other attached blocks, would be accepted in the Blockchain. The effort of a miner to obtain the optimal solution will result in the high probability of violations of the proposed and attached solutions. Figure 3 shows a schematic diagram of the reward function that indicates the relationship between the solution utility and mining efforts. The reward is designed for both maximization and minimization objective functions in Blockchain.

**Figure 4:**
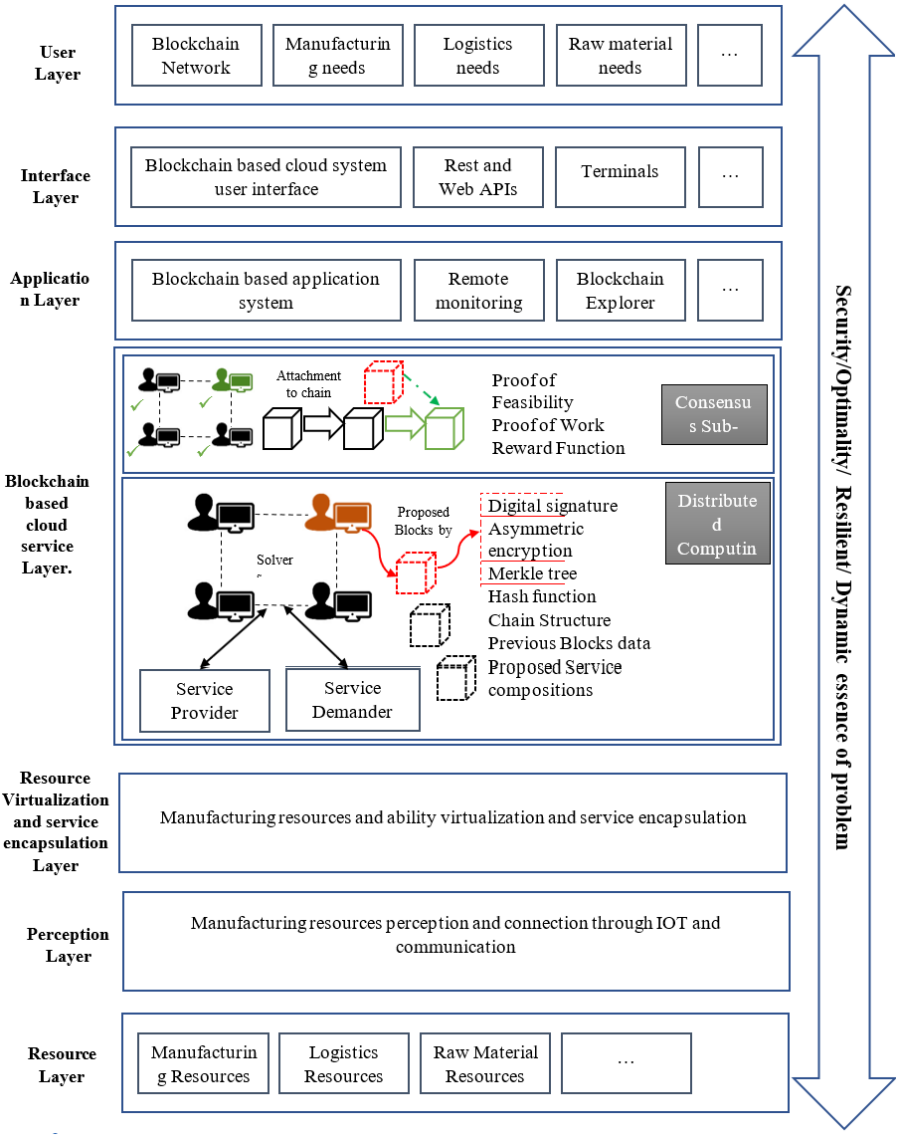
The blockchain-based cloud manufacturing platform.

If a miner has malicious purposes and wants to damage the system, the reward procedure will prevent it. The reward function is the most important barrier against potential malicious miners. In the proposed reward function, if a solver tends to propose a solution block with insufficient optimality, the given reward will proportionally be very low and not feasible in comparison with the service composition efforts. Also, the announcement process will not be logical and feasible in comparison with the commutating power. On the other hand, if a malicious miner wants to announce an infeasible solution block to the Blockchain, the consensus mechanism rejects the solution and the block cannot be attached to the Blockchain. In Blockchain, equitability is intrinsically not an issue. But the same access to data and resources is important and provides a shared ledger among the miners. In the proposed model, as described in Table 4 and illustrated in Fig. 4, each service demanders’ request and service providers’ capability are available in a shared pool, and solvers have equal access to resources. The aforementioned proposed mechanisms for blockchain-based cloud manufacturing system are embedded inside the blockchain based cloud layer inside the platform and enables the interactions of solvers with service providers and demanders.

**Table 4:** Blockchain concepts mapping with cloud manufacturing.

**Concept**	**Blockchain**	**Cloud manufacturing inspiration by blockchain**
**Transaction**	Each record of value transferring	Each suggested service/demand matching is represented as a transaction in blockchain.
**Block**	A set of valid transaction forms a block in blockchain.	A set of service/demand matchings forming a service composition solution that is relevant to a dedicated sub-problem constitute a block.
**Miner**	A miner is an agent who solves the mathematical puzzle to accomplish the consensus mechanism.	In proposed architecture solvers take the role of miners. Solvers try to solve the service demand matching sub-problems and propose a near-optimal solution and in turn achieve a correspondence reward.
**Consensus mechanism**	A mechanism to prevent malicious attacks in a distributed system.	Acceptance a solution for service composition in a sub-problem as a valid and sufficient good solution. It is considered as the proof of optimality. The allocated services in a proposed block should not violate the attached former blocks in the blockchain.
**Reward function**	A predefined reward to compensate miners’ efforts and guarantee continuity of the network.	The reward function is designed based on the optimality of the proposed solution. This relevance encourages miners to do their best for solving the service composition problem and announces near-optimal solutions.

Miners have two contradicting goals. On one hand, they want to create the optimal block by optimizing their proposed service compositions to achieve more rewards. On the other hand, they should be quick and agile in announcing their block to the network. Otherwise, another miner’s block might be accepted in the network instead and other miners’ effort be ineffectual. Also, as the dynamic behavior of service providers and demanders are realized in the system, the cloud will update the pool and solvers will response to the modified states in service providers’ and demanders’ attributes.

## Blockchain implementation considerations

The conceptual design mentioned in the previous section should be implemented through a blockchain platform such as Hyperledger or Ethereum. This paper has conducted a short analysis of the technical details and gives a holistic view of the proposed blockchain-based architecture. According to the literature (Wu et al., 2017), there are three types of Blockchain based on the miner’s level of access to the system and the information verification mechanism in blockchain:

1- Permissioned or private blockchain, where miners are known and transactions are approved by predetermined people.
2- Permission-less or public blockchain, where miners are unknown and everybody might confirm transactions and contribute to blockchain.
3- Hybrid blockchain, where both above models are used simultaneously in the system.

In this research, public blockchain is selected for the proposed model. Since the proposed problem assumes that the transacted information are not private and the goal is to accelerate finding the solution, therefore, public blockchain is selected to solve this problem. After introducing the basic concepts of blockchain, the mapping of a transaction is discussed. A block, a miner, a reward function, and a consensus mechanism are shown in Table 4. In this section, the technical elements in each transaction and block is described.

Public-key or asymmetric cryptography, which uses a pair of public and private keys, has been used in the various types of blockchain. A private key is produced by a random predefined mechanism. Then, the public key is created in a specific one-way hashing function. In such a cryptography system, any person can encrypt a message with the public key and decrypt it with the private key.

**Figure 5:**
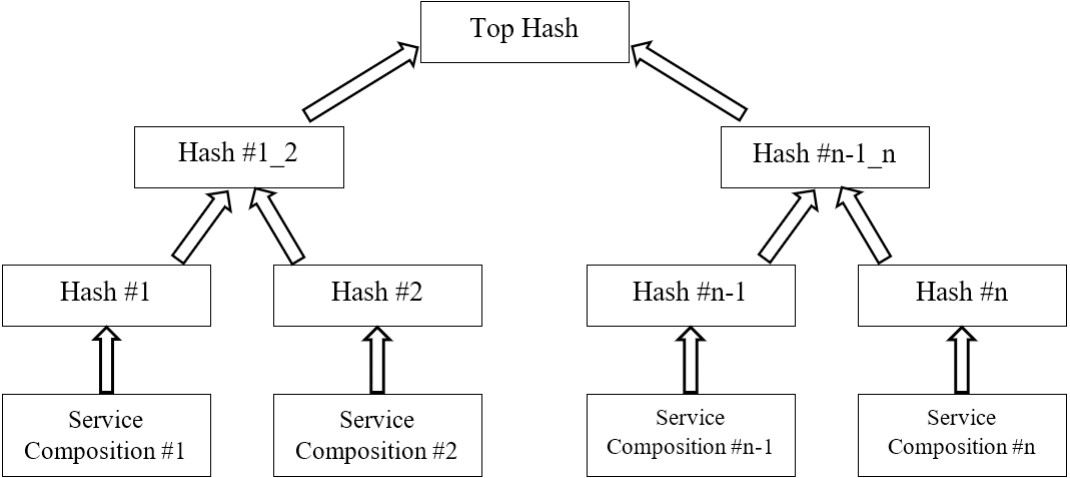
Cloud manufacturing top hash design in Merkle tree.

The Merkle tree is a good mechanism for compressing data and quickly investigating changes in a data set (Li et al., 2013). In Bitcoin whitepaper, the Merkle tree is used for hashing transaction information in a simple way. The Merkle tree used in the proposed software implementation helps simplify tracking changes and storing data. If a scammer wants to change the previous service compositions or impose his own preferences into the system, the Top Hash in the Merkle Tree would immediately change and other miners would know about the fraudulent changes (Fig. 5).

**Figure 6:**
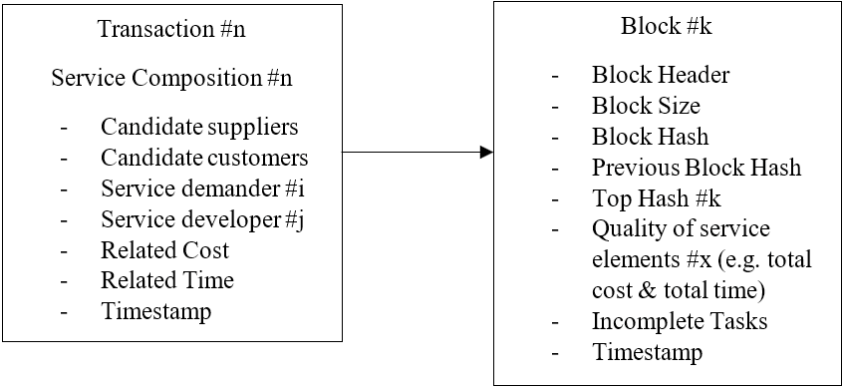
Cloud manufacturing transaction structure.

Each transaction consists of candidate suppliers and customers, the suggested service demander, relevant service developer, corresponding time and cost, and finally, the timestamp as shown in Fig. 6.

Each Block consists of the Merkle tree of transactions, block header, block hash, previous block hash, timestamps, etc. Each Block connects to its previous Block while storing the previous Block’s hash. This connectivity is an interpretation of the multi-period decision making in a supply chain. The previous decisions affect the next cost and time sub-problem.

## Numerical study for the capabilities of the proposed platform

In this section, a numerical study is presented for explaining the capabilities of the proposed blockchain-based cloud manufacturing model. The supposed cloud manufacturing system consists of enterprises as operational service providers and customers as service consumers considering the small and medium size industries in Tehran, Iran. Each service consumer has a set of tasks that can be operated by all the service providers. Different tasks of a service consumer can be allocated to various service providers. Therefore, a set of several enterprises perform the customer’s demand. The service cost and time for a specific task are not the same in different enterprises due to their capabilities and resource specifications. Also, locations of entities in the network, including enterprises and customers and the distances between them affect the transportation costs in the system.

In this case, tasks should be allocated to service providers in a way that results in the least amount of time and cost. So, the goal here is finding the service composition that generates the lowest cost. The cost function consists of processing and transportation costs for all of the tasks. The transportation cost is calculated based on the total distance traveled between service providers for delivering the half-finished products. The processing cost is different for each task and each service provider. This is an NP-hard problem and can be solved by combinatorial optimization algorithms. In the real world, the solving time is an essential factor for transforming a theoretical model to a practical environment. Therefore, in similar cases, achieving a proper solution in a sufficiently short time is more desirable than an optimal solution that needs excessive calculation time and infrastructure.

This paper has developed a genetic algorithm (GA), a well-known meta-heuristic algorithm used for solving this type of problem. Due to its ease of application and widespread application domain, many optimization problems use this meta-heuristic algorithm (Yang & Tinós, 2007).

**Figure 7:**
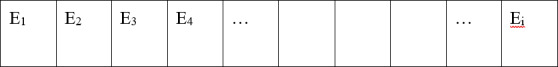
Chromosomes coding structure.

The main elements of the proposed genetic algorithm are explained here. Each chromosome is created from a set of natural numbers (Fig. 7). The total number of tasks represents the length of the chromosome string or the number of genomes. Each genome represents the corresponding task, and the numbers written on each genome represent the number of each enterprise. For example, if each demand consists of five tasks, the sixth genome represents the first task of the second demand. Moreover, each number in a genome is a delegate for a service provider enterprise where the defined task should be accomplished. For example, if number five is written on the sixth genome, it means the first task of the second demand will be fulfilled by the fifth enterprise.

**Figure 8:**
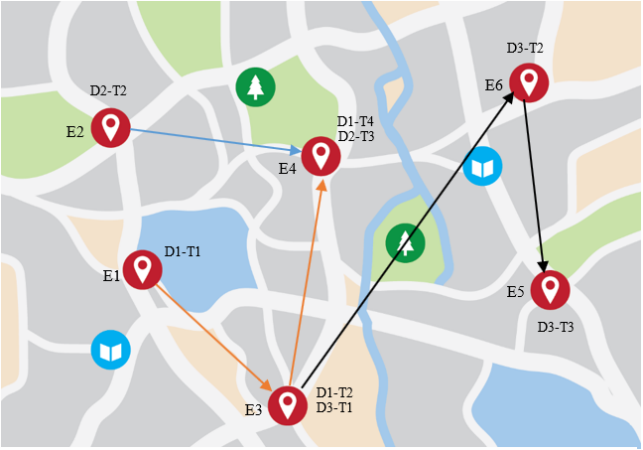
Illustration of cloud manufacturing service composition solution.

Two main operators in a genetic algorithm are crossover and mutation operators. The crossover operator empowers competitive selection in the genetic algorithm. Various types of crossover, such as uniform and two-point crossover, are proposed in the literature (Poon & Carter, 1995). This paper uses the two-point crossover. Another important operator in a genetic algorithm is the mutation that prevents hindering in a local optimum (Deb et al., 2002). For example, a cloud manufacturing system with six service providers and three demands is considered as a service composition solution. Each demand consists of a maximum of four tasks and all tasks can be accomplished by any service provider. Table 5 lists the required tasks for each demand. As explained before, the length of a chromosome is the total number of tasks, which, in this example, is eight. In the chromosome shown below, each cell or genome represents a task in relation to demand. Each number written in cells represents the corresponding service provider. In this example, the first task of the first demand would be completed in the first service provider, the second task in the third service provider, the fourth task in the fourth service provider, and so on. For this chromosome, the routes that each demand must take between service providers are shown in Fig. 8.

**Table 5:** Demands and the corresponding tasks in the numerical example and the corresponding chromosome configuration.

		Task 1	Task 2	Task 3	Task 4	
	Demand 1	✓	✓	–	✓	
	Demand 2	–	✓	✓	–	
	Demand 3	✓	✓	✓	–

D1-T1	D1-T2	D1-T4	D2-T2	D2-T3	D3-T1	D3-T2	D3-T3
1	3	4	2	4	3	6	5

**Figure 9:**
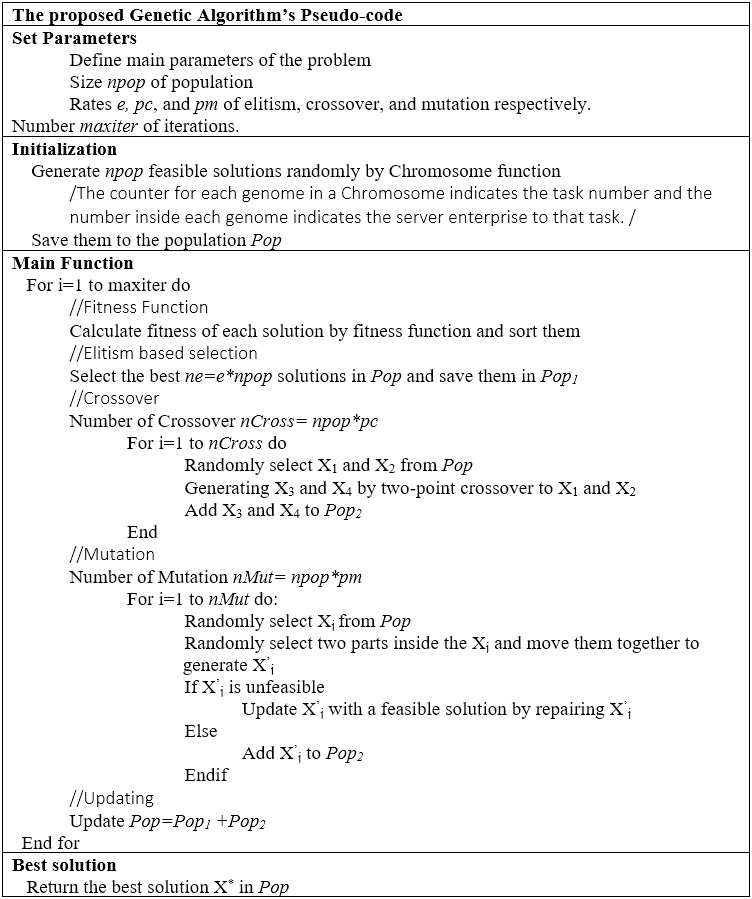
The pseudo-code of the proposed genetic algorithm.

The pseudo-code of the proposed genetic algorithm is shown in Fig. 9.

## Scenario 1: centralized mechanism

**Figure 10:**
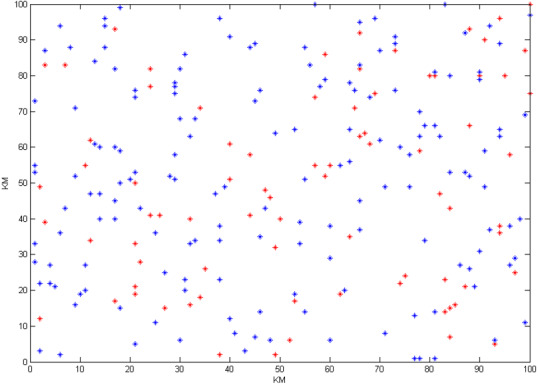
The geographical distribution of service providers and demanders in centralized mode.

**Figure 11:**
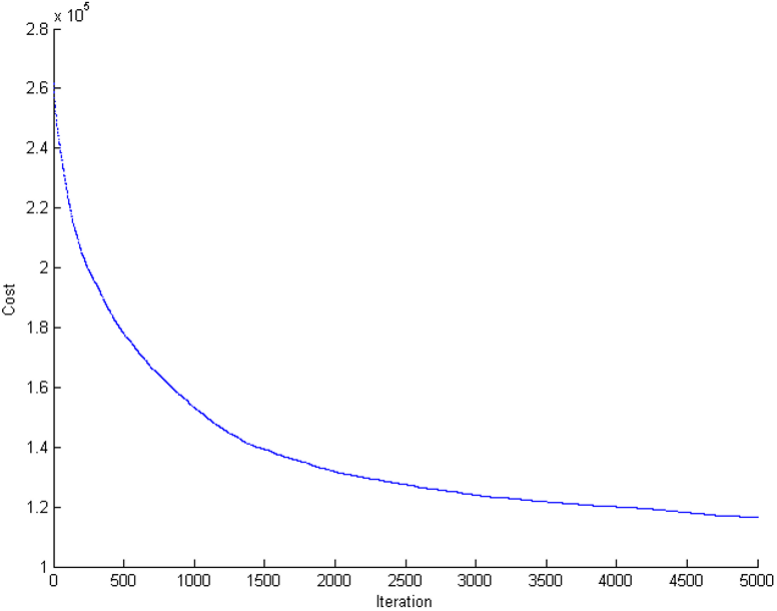
The convergence in objective function for supply chain configuration in centralized mode.

To illustrate the capabilities of the blockchain-based cloud manufacturing platform, this paper first solves the problem in the centralized model (traditional style). The manufacturing configuration problem is a mega-size problem. In this case, the problem has 50 service providers and 90 demanders, each of which has 25 orders. The assumed geographical distribution of nodes is an area of 100^2^ Km over the globe (Fig. 10). The problem is solved in the centralized model with the yielded objective function of 121,672 in the solving time of 68 s (Fig. 11). Since the cloud supply–demand service matching problem is a highly dynamic environment, the solving time is not acceptable in the real-world cases; demanders and service providers might change their service attributes and new demands and supplies might be added or removed during this period. This scenario will become worse as the problem grows in number of service providers, demanders, and tasks due to the NP-hard nature of the problem.

## Scenario 2: blockchain based supply–demand matching

**Figure 12:**
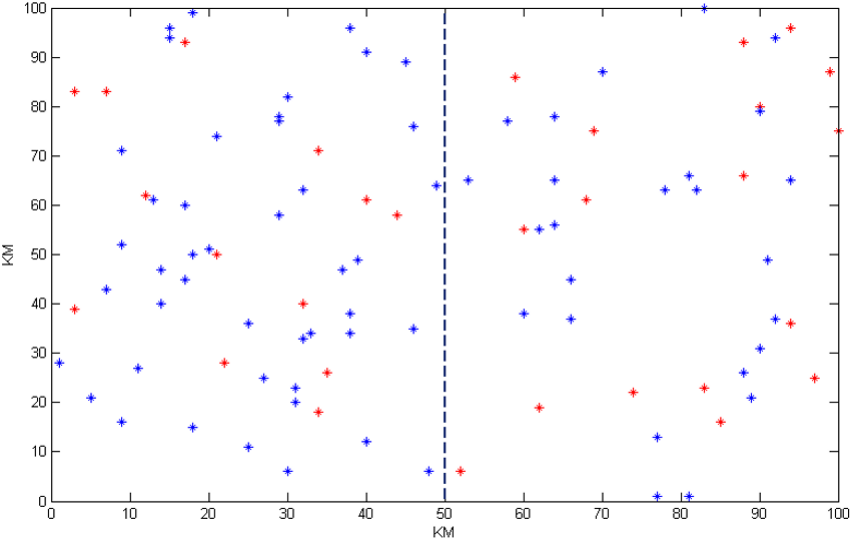
The geographical distribution of service providers and demanders for miners in blockchain-based mode.

In this scenario, the capabilities of the blockchain-based manufacturing system are used and it is assumed that only two miners start the process and configure the cloud manufacturing service composition. The conditions are the same as scenario 1; there are 50 service provider enterprises and 90 consumers with 25 demands each. The geographical distribution of nodes is in the same area of 100^2^ Km. In this scenario the miner can decide to select a sub-problem from the main problem. This paper assumes that the first miner selects a subset of the geographical area of 100 Km*50 Km covering 22 service provider enterprises and 40 service demanders. The second miner selects another subset of the geographical area of 100 Km*50 Km (the remaining part of the geographical area shown in Fig. 12) where the remaining 28 service provider enterprises and 50 service demanders are located. The details of the miners’ subset problems are shown in detail in Table 6. As the miners compete with each other, due to the smaller size of the sub-problems than the main problem, the first miner achieved the best solution of 44,001 in 38 s. At this point, the first miner announces his solution (supply–demand compositions) to the blockchain. The service composition set is accepted by the blockchain and will be appended. The second miner has started its progress at the same time (Table 6) after 54 s. The second miner announces its service composition set to the blockchain. As the first miner has announced their service composition set 16 s before, the blockchain should check for violations in this second set against the first miner’s attached set. In this scenario, the main problem has been divided into two distinct geographical areas. As shown in Fig. 12, the second miner’s solution does not violate any former service composition sets and it, too, will be attached to the blockchain.

 Each suggested service composition is stored in a different block. The new, refreshed conditions of the problem for demanders and service providers can be considered again for the next stages. The use of the Merkel tree mechanism makes it much easier to compare and validate answers from previous periods. Furthermore, each miner has a public and a private key to receive and expend their reward according to a cryptocurrency-based model. Also, asymmetric cryptography will increase the privacy of the system and will prevent the imposing of malicious preferences to the system.

In the proposed model, the consistency of data among various solving periods and the dynamic nature of the problem are assured and saved *via* the blockchain. The data in previous attached blocks will be considered for the attachment of the next block.

**Figure 13:**
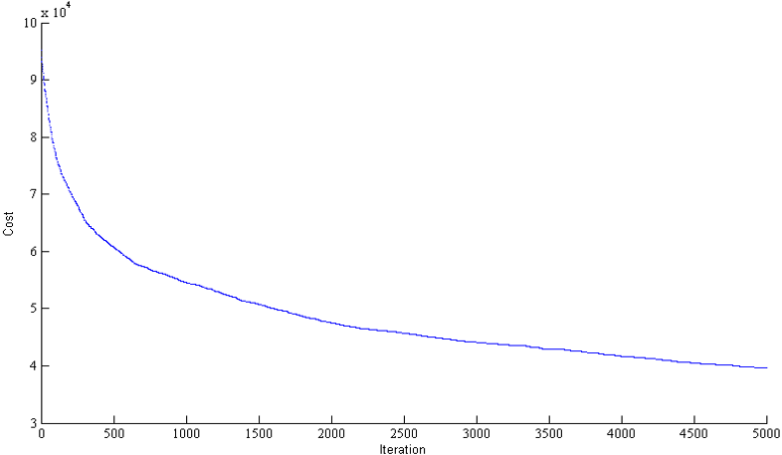
The convergence in objective function of the first miner for supply chain configuration in blockchain mode.

**Figure 14:**
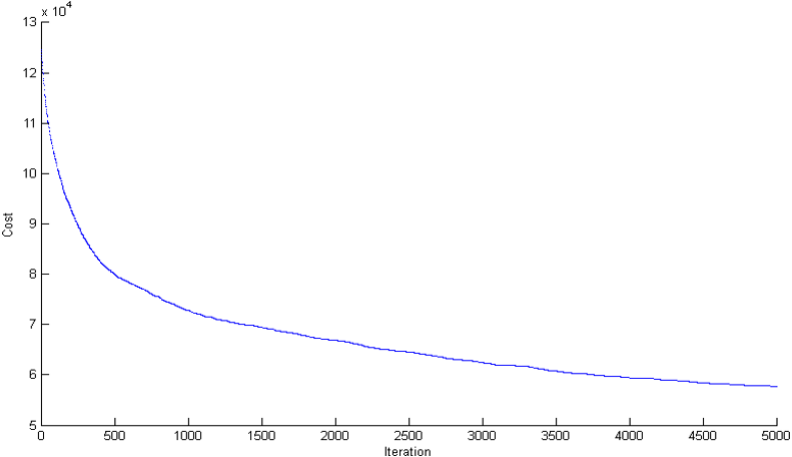
The convergence in objective function of the second miner for supply chain configuration in blockchain mode.

This paper tries to define the reward function as described in Table 6 by increasing the time efficiency in blockchain. This way, the rate of cancellation due to long waiting times can be dropped by 5%. Therefore, the mean revenue of service composition can be expected to increase by 5%. This paper assumes 8% of this increase as the maximum amount of reward (5%*121,672*8% = 486.68). Both miners achieved the maximum amount of reward due to the efficiency of their service composition algorithms. Since the second solver has solved the problem in 54 s, it is assumed as the longest time required to solve the problem. Compared to the required time in Scenario 1 (68 s), a 20% decrease is achieved. Also, the objective function of both solvers that represents the total objective function of the second solver equals 102515. Compared to the objective function in Scenario 1, a 15.74% improvement is achieved. As a result, Scenario 2 has provided a better solution both in terms of time and quality. The convergence of the miners’ algorithms has been illustrated in Figs. 13 and 14.

**Table 6:** The scenarios specifications in both centralized and blockchain-based models.

	*Scenario 1*	*Scenario 2*	
*Mode*	*Centralized*	*Blockchain based*	
		*Miner#1*	*Miner#2*
*Service provider enterprises*	*50*	*22*	*28*
*Service demanders*	*90*	*40*	*50*
*Number of orders for each demander*	*25*	*25*	*25*
*Geographical area dimension*	100 Km*100 Km	100 Km*50 Km	100 Km*50 Km
*Solving time*	*762 s*	*540 s*	*551 s*
*The yielded objective function cost*	*116,410*	*35,598*	*57,675*
*The reward function*	*n/a*	$= \left\{ \begin{array}{@{}l@{}} \displaystyle 465.64 OF\leq 58,205\\ \displaystyle ( \frac{-465.64}{\pi } \ast {\cot }^{-1}(.0028\ast OF-165.33)) OF\gt 58,205 \end{array} \right. $ *OF:miner announced Objective Function*	
*The best expected objective function*		*58,205*	*58,205*
*The yield reward for the miner*		*465.64*	*465.64*

**Figure 15:**
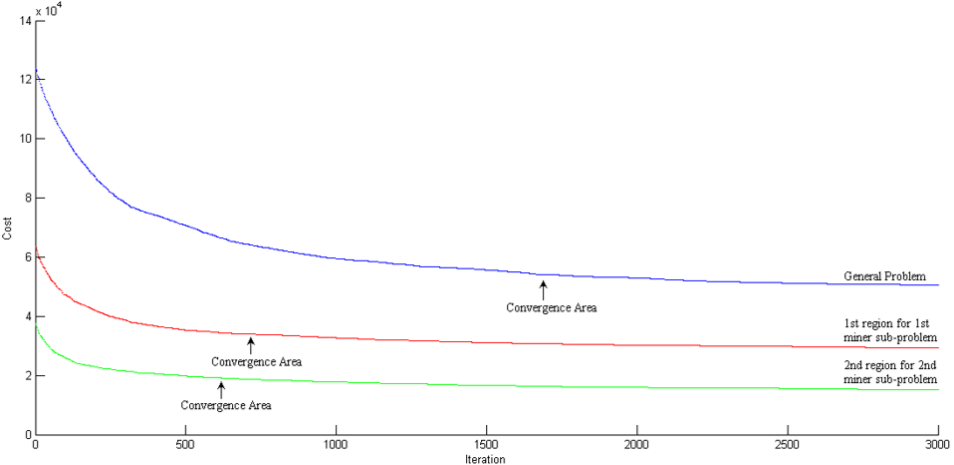
The comparison among the blockchain and centralized service composition algorithms convergence.

The results are promising, as both the blockchain-based platform objective functions and the time for service composition set configuration are improved. This is justified as the problem is of an NP-hard nature and meta-heuristic approaches are applied in both scenarios. In the blockchain-based scenario, the miners are dealing with smaller sub-problems. As a result, both the solving time and the quality of the solution can increase (Fig. 15). Furthermore, as the cloud supply–demand service matching problem has a highly dynamic environment, engaging more miners allows them to improve the solving cycle efficiency.

In order to examine the results in more detail, three test problems with different dimensions and considering the 3 and 4 solvers (miners) modes have been studied. Table 7 shows the specifications of each of the 4 problems in full, and Table 8 shows the output of the solution on each problem. As previously stated, in distributed cases the problem-solving time is equal to the longest solving time by each miner and the problem cost is equal to the total cost of solving each miner. In the final column, the amount of improvement created between the concentrated states and the different distributed states is examined in terms of solution time and solution cost.

**Table 7:** Test problems’ parameters.

Parameters	Problem 1	Problem 2	Problem 3	Problem 4
Service provider enterprises	30	50	60	90
Service demanders	70	90	140	180
Number of orders for each demander	15	25	20	40
Geographical area dimension	100 Km*100 Km	100 Km*100 Km	100 Km*100 Km	200 Km*200 Km

**Table 8:** Comparison expressed problems solving.

	Centralized		Blockchain Based, 2 Solvers		Blockchain based, 3 Solvers		Blockchain based, 4 Solvers		Improvement (%)						
	Cost ($)	Solving time (sec)	Cost ($)	Solving time (sec)	Cost ($)	Solving time (sec)	Cost ($)	Solving Ttime (sec)	Centralized *vs.* BB 2 Solvers		Centralized *vs.* BB 3 Solvers		Centralized *vs.* BB 4 Solvers		
									Cost	Time	Cost	Time	Cost	Time	
Problem 1	53748	39	45608	27.5	42159	23.5	40490	20	15.14	29.4	21.56	39.7	24.67		48.7
Problem 2	121672	68	102515	54	87812	39.4	86609	28.1	15.74	20	27.8	42	28.8		58. 7
Problem 3	164611	83.7	135689	47	118133	39.9	107302	38.3	17.56	43.8	28.2	52.3	34.8		54.2
Problem 4	610577	198.1	499584	124.1	422786	101.7	415526	62.6	18.17	37.4	30.76	48.66	31.94		68.4

As can be seen in Table 8, in different cases the blockchain-based solution has better solutions than the centralized solution in terms of solution time and cost. In problem number 4, which is the largest problem under study, the answer in 4-solver mode compared to the centralized mode has a 31.94% improvement in cost and 68.4% improvement in solving time, which means high efficiency of the proposed model, especially in large scale problems.

**Figure 16:**
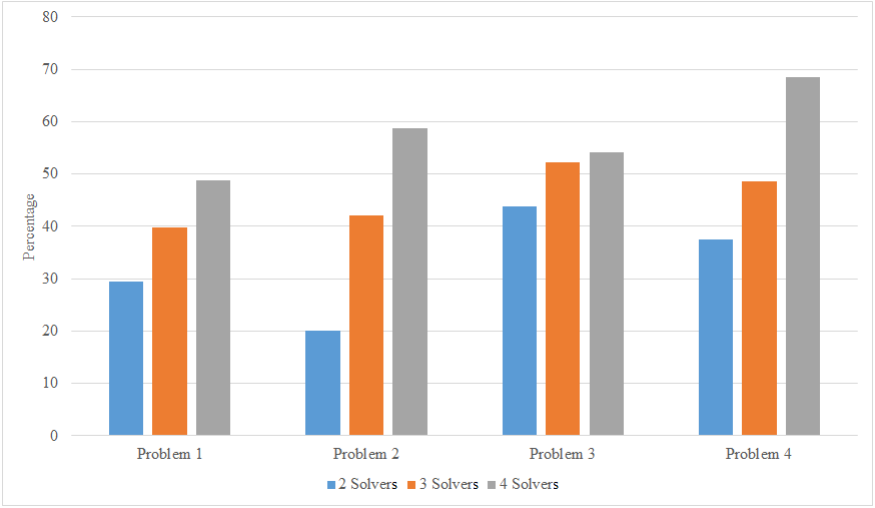
Comparison between number of solvers and percentage of solving time improvement.

As can be seen in Fig. 16, the percentage of improvement made during problem solving increases with the number of solvers in different problems. It can be concluded that the more a problem is solved in a more distributed way, the faster the response will be without negatively affecting the final answer. Finally, the results have been compared in Table 9 with the recent studied literature in Table 2 to compare the performance and also the capabilities of the proposed solution. The comparison is considered based on the dominant factors related to the issue of service composition, including the optimality, and maintaining a competitive advantage among actors. The results indicate that the proposed model improves the optimality of provided solutions although using the meta-Heuristic approach this is due to the provided reward function mechanism. Previous research studies have not focused on distributed solving mechanism by blockchain in terms of competitive advantage security which in this paper hash mechanism it is also fulfilled.

**Table 9:** Comparison of the proposed method performance with the current studied literature.

Reference	Optimality of provided solution	Existence of reward function to maintain a stable relationship	Decentralization in solution finding mechanism	Maintain the competitive advantage of the actors (Encryption and non- disclosure of information)
Hasan & Starly (2020)	N/A In this article, only the proposed architecture and its implementation have been introduced and the issue of problem solving has not been studied.	The concept of miners has been introduced but miner has not been used as a solver.	The issue of problem solving has not been addressed.	In general, the cryptographic process is used.
Yu et al. (2020)	Due to the NP-Hard problem the meta-Heuristic solution presented, the optimal answer has not been guaranteed and only the quality of the answers has been examined.	Just a general introduction	The issue of problem solving has not been addressed.	In general, the cryptographic process is used.
Aghamohammadzadeh & Valilai (2020)	N/A The problem has been studied only in terms of architecture and has not been solved numerically	Just a general introduction	It is generally stated in the proposed architecture	In general, the cryptographic process is used.
Zhu et al. (2020a) and Zhu et al. (2020b)	N/AA different issue (pricing issue) has been explored in the cloud manufacturing space	NO	NO	NO
Vatankhah Barenji (2021)	N/A The problem has been studied only in terms of architecture and has not been solved numerically	NO	NO	NO
Zhang et al. (2021)	N/A The problem has been studied only in terms of architecture and has not been solved numerically	Yes. A new consensus mechanism and a corresponding reward function have been introduced at the architectural level	It is generally stated in the proposed architecture	In general, the cryptographic process is used.
Zhu et al. (2020a) and Zhu et al. (2020b)	N/A The problem has been studied only in terms of architecture and has not been solved numerically	Yes. A new consensus mechanism and a corresponding reward function have been introduced at the architectural level	It is generally stated in the proposed architecture	In general, the cryptographic process is used.
Barenji, Li & Wang (2018), Vatankhah Barenji et al. (2018), Vatankhah Barenji et al. (2020)	N/A The problem has been studied only in terms of architecture and has not been solved numerically	NO	NO	NO
Li et al. (2019)	N/A The problem has been studied only in terms of architecture and has not been solved numerically	NO	NO	NO
Proposed model	The proposed model has been tested in four problems. The proposed solution will lead to at least 15.14% and a maximum of 34.8 percent reduction in costs and 20 to 68.4 percent at the solving time of the problem.	YES, the mechanisms have been discussed in operational level. It is also designed to increase the optimality of main problem.	YES, the mechanisms have been discussed in operational level.	YES, the hash mechanism has been introduced to secure the matching among service providers and service demanders.

## Discussion and conclusion

The paradigms like cloud manufacturing and cyber-physical systems have resulted in new Manufacturing network ecosystems for disruptive service-oriented business models. A service-oriented architecture is one of the main components of the cloud platform. As stated by the XaaS paradigm in cloud manufacturing, the agents interact by exchanging services with each other. This makes the service composition research topic one of the most important issues in cloud manufacturing. Due to the dynamic behavior of agents in cloud manufacturing, research studies on service compositions and their allocation are challenging. This paper suggests the essential need for a dynamic approach in service composition mechanisms.

This paper has reviewed the current literature on the cloud-based service composition and has discussed the inefficiency of the centralized global optimization models in fulfilling the dynamic conditions of cloud manufacturing. Considering this fact and the size of problems in globalized cloud manufacturing, this paper proposes a distributed service composition enabler platform. This platform uses blockchain technology, which is an innovative disruptive technology that can solve problems of operation management in industrial applications. In blockchain, each service-demand composition is considered as a transaction. A block consists of a service composition set and is validated by a predefined consensus mechanism in the manufacturing network. The platform considers the miners’ roles competing for proposing service composition solutions and attaching them to the blocks. Moreover, this paper has designed the major components of the blockchain-based platform, including transaction, blocks, miner roles, consensus mechanism, and reward function.

The capabilities of the proposed platform were investigated *via* a numerical study. It was found that, although it approaches sub-problems, the blockchain-based approach increases the optimality of the overall service compositions. This ability is achieved as miners deal more efficiently with the NP-hard nature of the problems, resulting in better service composition sets. Moreover, dealing with sub-problems, the miners announce their optimal service compositions sets in a considerably shorter time. This results in the increased efficiency of the manufacturing network and its capabilities for fulfilling the dynamic behavior of service providers and demanders. The authors recommend the following research topics for future research and studies:

• The idea of a blockchain manufacturing system can be extended based on the architecture of the blockchain technology. The special recommendation is designing consensus mechanisms which can enable the confirmation for service composition modifications. This paper has considered the service capabilities to be dedicated. However, it is strongly recommended to consider the partial allocation structure of services, and that the miners use the partially allocated services in former blocks to improve optimality. This requires the application and redefinition of the concept of block confirmation.
• In this paper, geographical attributes are used to enable the miners in defining sub-problems. It is strongly recommended that ideas and models are investigated to help the miners select their sub-problems based on various attributes in the cloud manufacturing system.

## Additional Information and Declarations

### Data Availability

The following information was supplied regarding data availability: The sample data for big and mega size problems and codes for solving tasks are available at figshare: - Fatahi Valilai, Omid (2021): Big size data. figshare. Dataset. https://doi.org/10.6084/m9.figshare.14247236.v1
- Fatahi Valilai, Omid (2021): Untitled Item. figshare. Dataset. https://doi.org/10.6084/m9.figshare.14247245.v1
- Fatahi Valilai, Omid (2021): DataSet and Codes for Blockchain based Cloud manufacturing. figshare. Dataset. https://doi.org/10.6084/m9.figshare.14225954.v1.

## References

[ref-1] Adamson G, Wang L, Holm M, Moore P (2017). Cloud manufacturing–a critical review of recent development and future trends. International Journal of Computer Integrated Manufacturing.

[ref-2] Aghamohammadzadeh E, Malek M, Valilai OF (2019). A novel model for optimisation of logistics and manufacturing operation service composition in Cloud manufacturing system focusing on cloud-entropy. International Journal of Production Research.

[ref-3] Aghamohammadzadeh E, Valilai OF (2020). A novel cloud manufacturing service composition platform enabled by Blockchain technology. International Journal of Production Research.

[ref-4] Ahmadi E, Khaturia R, Sahraei P, Niyayesh M, Valilai OF (2021). Using blockchain technology to extend the vendor managed inventory for sustainability.

[ref-5] Antonopoulos AM (2014). Mastering Bitcoin: unlocking digital cryptocurrencies.

[ref-6] Apte S, Petrovsky N (2016). Will blockchain technology revolutionize excipient supply chain management?. Journal of Excipients and Food Chemicals.

[ref-7] Barenji AV, Li Z, Wang WM (2018). Blockchain cloud manufacturing: shop floor and machine level.

[ref-8] Bouzary H, Chen FF (2019). A hybrid grey wolf optimizer algorithm with evolutionary operators for optimal QoS-aware service composition and optimal selection in cloud manufacturing. The International Journal of Advanced Manufacturing Technology.

[ref-9] Crosby M, Pattanayak P, Verma S, Kalyanaraman V (2016). Blockchain technology: beyond bitcoin. Applied Innovation.

[ref-10] Deb K, Pratap A, Agarwal S, Meyarivan T (2002). A fast and elitist multiobjective genetic algorithm: NSGA-II. IEEE Transactions on Evolutionary Computation.

[ref-11] Delaram J, Houshamand M, Ashtiani F, Valilai OF (2021a). A utility-based matching mechanism for stable and optimal resource allocation in cloud manufacturing platforms using deferred acceptance algorithm. Journal of Manufacturing Systems.

[ref-12] Delaram J, Valilai OF (2016). Development of a novel solution to enable integration and interoperability for cloud manufacturing. Procedia CIRP.

[ref-13] Delaram J, Valilai OF (2017). A novel solution for manufacturing interoperability fulfillment using interoperability service providers. Procedia CIRP.

[ref-14] Delaram J, Valilai OF (2018). An architectural view to computer integrated manufacturing systems based on axiomatic design theory. Computers in Industry.

[ref-15] Delaram J, Valilai OF, Houshamand M, Ashtiani F (2021b). A matching mechanism for public cloud manufacturing platforms using intuitionistic Fuzzy VIKOR and deferred acceptance algorithm. International Journal of Management Science and Engineering Management.

[ref-16] Fazeli MM, Farjami Y, Nickray M (2019). An ensemble optimisation approach to service composition in cloud manufacturing. International Journal of Computer Integrated Manufacturing.

[ref-17] Francisco K, Swanson D (2018). The supply chain has no clothes: technology adoption of blockchain for supply chain transparency. Logistics.

[ref-18] Garay J, Kiayias A (2020). Sok: a consensus taxonomy in the blockchain era.

[ref-19] Hasan M, Starly B (2020). Decentralized cloud manufacturing-as-a-service (CMaaS) platform architecture with configurable digital assets. Journal of Manufacturing Systems.

[ref-20] Helo P, Hao Y (2019). Blockchains in operations and supply chains: a model and reference implementation. Computers & Industrial Engineering.

[ref-21] Houshmand M, Valilai OF (2013). A layered and modular platform to enable distributed CAx collaboration and support product data integration based on STEP standard. International Journal of Computer Integrated Manufacturing.

[ref-22] Iansiti M, Lakhani KR (2017). The truth about blockchain. Harvard Business Review.

[ref-23] Kim HM, Laskowski M (2018). Toward an ontology-driven blockchain design for supply-chain provenance. Intelligent Systems in Accounting, Finance and Management.

[ref-24] Lakshman T, Agrawala AK (1986). Efficient decentralized consensus protocols. IEEE Transactions on Software Engineering.

[ref-25] Li R, Chen T, Lou P, Yan J, Hu J (2019). Trust mechanism of cloud manufacturing service platform based on blockchain.

[ref-26] Li H, Lu R, Zhou L, Yang B, Shen X (2013). An efficient merkle-tree-based authentication scheme for smart grid. IEEE Systems Journal.

[ref-27] Li F, Zhang L, Liu Y, Laili Y, Tao F (2017). A clustering network-based approach to service composition in cloud manufacturing. International Journal of Computer Integrated Manufacturing.

[ref-28] Liu Y, Xu X (2017). Industry 4.0 and cloud manufacturing: a comparative analysis. Journal of Manufacturing Science and Engineering.

[ref-29] Manenti P (2011). Building the global cars of the future. Managing Automation.

[ref-30] Meixell MJ, Gargeya VB (2005). Global supply chain design: a literature review and critique. Transportation Research Part E: Logistics and Transportation Review.

[ref-31] Nakamoto S (2008). Bitcoin: a peer-to-peer electronic cash system.

[ref-32] Poon PW, Carter JN (1995). Genetic algorithm crossover operators for ordering applications. Computers & Operations Research.

[ref-33] Ren L, Zhang L, Tao F, Zhao C, Chai X, Zhao X (2015). Cloud manufacturing: from concept to practice. Enterprise Information Systems.

[ref-34] Rezapour Niari M, Eshgi K, Fatahi Valilai O (2021). Topology analysis of manufacturing service supply–demand hyper-network considering QoS properties in the cloud manufacturing system. Robotics and Computer-Integrated Manufacturing.

[ref-35] Sankar LS, Sindhu M, Sethumadhavan M (2017). Survey of consensus protocols on blockchain applications.

[ref-36] Seebacher S, Schüritz R (2017). Blockchain technology as an enabler of service systems: a structured literature review.

[ref-37] Strandhagen JO, Vallandingham LR, Fragapane G, Strandhagen JW, Stangeland ABH, Sharma N (2017). Logistics 4.0 and emerging sustainable business models. Advances in Manufacturing.

[ref-38] Supriya BA, Djearamane I (2013). RFID based cloud supply chain management. International Journal of Scientific & Engineering Research.

[ref-39] Tong H, Zhu J (2020). A two-layer social network model for manufacturing service composition based on synergy: a case study on an aircraft structural part. Robotics and Computer-Integrated Manufacturing.

[ref-40] Valilai OF, Houshmand M (2014a). Depicting additive manufacturing from a global perspective; using Cloud manufacturing paradigm for integration and collaboration. Proceedings of the Institution of Mechanical Engineers, Part B: Journal of Engineering Manufacture.

[ref-41] Valilai OF, Houshmand M (2014b). A manufacturing ontology model to enable data integration services in cloud manufacturing using axiomatic design theory. Cloud-based design and manufacturing.

[ref-42] Valilai OF, Houshmand M (2014c). A platform for optimisation in distributed manufacturing enterprises based on cloud manufacturing paradigm. International Journal of Computer Integrated Manufacturing.

[ref-43] Vatankhah Barenji R (2021). A blockchain technology based trust system for cloud manufacturing. Journal of Intelligent Manufacturing.

[ref-44] Vatankhah Barenji A, Guo H, Tian Z, Li Z, Wang WM, Huang GQ (2018). Blockchain-based cloud manufacturing: decentralization.

[ref-45] Vatankhah Barenji A, Li Z, Wang WM, Huang GQ, Guerra-Zubiaga DA (2020). Blockchain-based ubiquitous manufacturing: a secure and reliable cyber-physical system. International Journal of Production Research.

[ref-46] Wang Y, Han JH, Beynon-Davies P (2019). Understanding blockchain technology for future supply chains: a systematic literature review and research agenda. Supply Chain Management: An International Journal.

[ref-47] Wu L, Meng K, Xu S, Li S, Ding M, Suo Y (2017). Democratic centralism: a hybrid blockchain architecture and its applications in energy internet.

[ref-48] Yang S, Tinós R (2007). A hybrid immigrants scheme for genetic algorithms in dynamic environments. International Journal of Automation and Computing.

[ref-49] Yang Y, Yang B, Wang S, Liu F, Wang Y, Shu X (2019a). A dynamic ant-colony genetic algorithm for cloud service composition optimization. The International Journal of Advanced Manufacturing Technology.

[ref-50] Yang Y, Yang B, Wang S, Liu W, Jin T (2019b). An improved grey wolf optimizer algorithm for energy-aware service composition in cloud manufacturing. The International Journal of Advanced Manufacturing Technology.

[ref-51] Yu C, Zhang L, Zhao W, Zhang S (2020). A blockchain-based service composition architecture in cloud manufacturing. International Journal of Computer Integrated Manufacturing.

[ref-52] Yuan M, Zhou Z, Cai X, Sun C, Gu W (2020). Service composition model and method in cloud manufacturing. Robotics and Computer-Integrated Manufacturing.

[ref-53] Zhang L, Guo H, Tao F, Luo Y, Si N (2010). Flexible management of resource service composition in cloud manufacturing.

[ref-54] Zhang W, Yang Y, Zhang S, Yu D, Li Y (2018). Correlation-aware manufacturing service composition model using an extended flower pollination algorithm. International Journal of Production Research.

[ref-55] Zhang Y, Zhang L, Liu Y, Luo X (2021). Proof of service power: a blockchain consensus for cloud manufacturing. Journal of Manufacturing Systems.

[ref-56] Zhou J, Yao X (2017). A hybrid approach combining modified artificial bee colony and cuckoo search algorithms for multi-objective cloud manufacturing service composition. International Journal of Production Research.

[ref-57] Zhou J, Yao X, Lin Y, Chan FT, Li Y (2018). An adaptive multi-population differential artificial bee colony algorithm for many-objective service composition in cloud manufacturing. Information Sciences.

[ref-58] Zhu X, Shi J, Huang S, Zhang B (2020a). Consensus-oriented cloud manufacturing based on blockchain technology: an exploratory study. Pervasive and Mobile Computing.

[ref-59] Zhu X, Shi J, Xie F, Song R (2020b). Pricing strategy and system performance in a cloud-based manufacturing system built on blockchain technology. Journal of Intelligent Manufacturing.

